# Trends and determinants of vaccination among children aged 06–59 months in Bangladesh: country representative survey from 1993 to 2014

**DOI:** 10.1186/s12889-021-11576-0

**Published:** 2021-08-21

**Authors:** Md. Moyazzem Hossain, Md. Abdus Sobhan, Azizur Rahman, Sanzida Sharmin Flora, Zahida Sultana Irin

**Affiliations:** 1 Department of Statistics, Jahangirnagar University, Savar, Dhaka, 1342 Bangladesh; 2 School of Mathematics, Statistics & Physics, Newcastle University, Newcastle upon Tyne, UK; 3 Chief Economist’s Unit, Bangladesh Bank, Head Office, Dhaka, 1000 Bangladesh; 4 School of Computing, Mathematics and Engineering, Charles Sturt University, Wagga Wagga, NSW 2678 Australia; 5 Ex-Indoor Medical Officer, Islami Bank Hospital, Mirpur, Dhaka, 1000 Bangladesh; 6 Department of Medicine, University of British Columbia, Vancouver, V5Z 1M9 British Columbia Canada

**Keywords:** Trends in coverage rate, Vaccination, Adjusted logistic regression, BDHS, Bangladesh

## Abstract

**Background:**

Vaccination has important consequences for childhood development, mortality, and inequalities in health and well-being. This research explores the trend of vaccinations coverage from 1993 to 2014 and determines the significant factors for vaccinations coverage in Bangladesh, considering geospatial, socioeconomic, and demographic characteristics.

**Methods:**

This study uses a secondary dataset extracted from the Bangladesh Demographic and Health Survey (BDHS) from 1992 to 93 to 2014. The association between selected independent variables and vaccination coverage of children was examined through the Chi-square test. In addition, unadjusted and adjusted logistic regression approaches were applied to determine the effects of covariates on vaccination status by using the BDHS-2014 dataset.

**Results:**

The results reveal that the trend of the vaccination coverage rate has gradually been increased over the study period. The coverage rate of BCG is observed maximum while the lowest for Measles vaccination among all types of vaccinations. The findings revealed that the significantly lower coverage of all vaccination had been observed in the Sylhet region. Children of higher educated mothers (OR 10.21; CI: 4.10–25.37) and father (OR 8.71; CI: 4.03–18.80), born at health facilities (OR 4.53; CI: 2.4–8.55) and whose mother has media exposure (OR 3.20; CI: 2.22–4.60) have more chance of receiving BCG vaccine. For DPT vaccination coverage, there is a significant difference from children whose mothers have primary (OR 1.7; CI: 1.35–2.15), secondary (OR 3.5; CI: 2.75–4.45), and higher (OR 9.6; CI: 5.28–17.42) educational qualification compared to children of illiterate mothers. Findings demonstrated that children born in wealthier households have a higher likelihood of being immunized against DPT, Polio, and Measles vaccination than children born in the poorest households.

**Conclusions:**

The findings reveal that to enhance and make sustainable the overall country’s vaccination coverage, we should pay more attention to the mother’s education, socioeconomic condition, children’s age, birth order number, having media exposure, place of residence, and religion. The authors think that this finding would be helpful to accelerate the achievement target of Sustainable Development Goals (SDGs) for children’s health in Bangladesh.

**Supplementary Information:**

The online version contains supplementary material available at 10.1186/s12889-021-11576-0.

## Background

The Expanded Program on Immunization (EPI) was officially established in 1974, with the support of the World Health Organization (WHO), keeping in mind as an important goal of immunizing every child counter to four vaccine-preventable diseases (VPD) (i.e., tuberculosis, poliomyelitis, measles and diphtheria, pertussis, tetanus (DPT)) by 1990 [1]. However, the EPI set the priority for the developing countries since the higher prevalence and inadequate service delivery for immunization are observed in those countries [2]. The immunization programme for children against VPD has been considered one of the best cost-effective programmes to lessen childhood morbidities and mortalities throughout the world [3, 4]. Moreover, vaccination against VPDs averts illness and disability, saving millions of children globally every year [5]. The rates of vaccine-preventable diseases have declined in many regions worldwide in the past few decades, but many children remain unvaccinated [6]. Moreover, every year, nearly 20 million infants do not receive the complete set of recommended vaccines globally [7]. However, over the past decades, a remarkable improvement has been observed in developing the national immunization program [8]. According to a recent study, the supply chain of lifesaving vaccination has lately been disrupted in several nations due to the lockdown measures adopted by most countries due to COVID-19 [9]. 

Vaccines have saved uncountable lives, minimized the incidence of polio globally by 99% and reduced illness, disability, and death from several diseases like tetanus, diphtheria, measles, *H. influenzae* type b disease, whooping cough and epidemic meningococcal A meningitis [10]. Therefore, immunization should be considered a fundamental element of the human right to health and must play a vital role by the individual, community, and government of a country. Like other countries, the government of Bangladesh had originated EPI with the backing of UNICEF and WHO via several activities from 1979, with the overall objective to immunize all children by 1990 to prevent the VPDs and eliminate poliomyelitis [1, 11]. As a result, Bangladesh has experienced notable enhancements in increasing immunization coverage and a noteworthy contribution to the lessening of childhood morbidity, mortality and also continuing its polio-free status [12]. However, despite declining the child mortality rate, the country included in the list of the top ten countries with the highest childhood mortality globally [13].

In several countries, it has been identified that vaccination coverage is associate with socioeconomic and demographic characteristics. Previous studies have been reported that parental education level [4, 14–17], age of child [16, 18, 19], employment status as well as workplace [14, 16, 17], religion [20], ethnic origin [21], sex of child [22], poverty [23–27], and distance to healthcare facilities [14, 16, 20, 24, 26, 27] are linked to vaccination coverage. However, significant differences often exist between countries [20]. Moreover, the findings of several previous studies have generated evidence about the socio-demographic factors related to full vaccination among children [28–30]. Furthermore, several existing studies have focused on specified geographical settings, age groups, or ethnic groups rather than the countrywide setting and have not identified the determinants of individual vaccines separately [2, 3, 29–34]. The authors were motivated to fill the gap of finding out the potential determinants of individual vaccination coverage of BCG, DPT, Polio, and Measles in Bangladesh. Therefore, this study demonstrates the trend from 1993 to 2014 and sought out the influential determinant to capture the full vaccination status using the 2014 Bangladesh Demographic and Health Survey (BDHS-2014) dataset.

## Methods

### Data sources, study design and participants

To bear in mind the first objectives of this study, i.e., for trend estimation purposes, the authors used seven nationally representative Bangladesh Demographic Health Survey (BDHS) data (1993–1994, 1996–1997, 1999–2000, 2004, 2007, 2011 and 2014) and finally, to identify the significant determinants of child vaccination coverage against BCG, DPT, Polio and Measles, this study uses the BDHS-2014 data set. In BDHS-2014, the sampling frame was used as a complete list of enumeration areas (EAs) covering the country prepared by the Bangladesh Bureau of Statistics for the 2011 population census of the People’s Republic of Bangladesh. An EA is a geographic area covering, on average, 113 households. However, the 2014 BDHS sample was stratified and selected in two stages. In the first stage, 600 EAs were selected with probability proportional to the EA size. A fixed number of 30 households per cluster was selected in the second stage selection with an equal probability from the newly created household listing. The data sets were filtered with the inclusion criteria of infants aged between six and 59 months. In addition, the small amount of system missing observations was discarded from the dataset. In order to find out the trend of vaccinations mentioned above, the number of children was 2387, 4468, 5117, 5220, 4614, 6715 and 6246 for BDHS surveys 1993–1994, 1996–1997, 1999–2000, 2004, 2007, 2011 and 2014 respectively. However, the findings of the subsequent analysis were based on 6246 children. The detailed data collection procedures are available on the report of the respective years [35–41].

### Study variables

This study considers four vaccination, i.e., BCG, DPT, Polio, and Measles coverage in Bangladesh. The outcome variable of interest in this study is whether a child received the selected vaccination or not. In this case, if a child received the vaccine, then it is coded as “1” represents “yes”, and otherwise, it is coded as “0” indicates “no”. Several demographic, socioeconomic and spatial variables were included in the analysis as predictors of a child’s vaccination status. The predictor variables include place of residence (urban or rural), region (Barisal, Chittagong, Dhaka, Khulna, Rajshahi, Rangpur, and Sylhet), religion (Muslim or Non-Muslim), mother’s age, mother’s education level (no education, primary, secondary and higher) and working status (no, yes), father’s education level (no education, primary, secondary and higher) and occupation (farming, non-farming, professionals, business, unemployed and others), and wealth index (poorest, poorer, middle, richer, and richest). The selection of variables used in this study was motivated by the availability in the BDHS dataset and self-efficacy and guided by relevant literature. The Expanded Program on Immunizations (EPI) schedule and timeliness in Bangladesh are given in Table 1.

**Table 1:** Schedule of the Expanded Program on Immunizations in Bangladesh

Vaccine	Recommended age period
Bacille Calmette Guerin (BCG)	At birth/0 day
Pentavalent 1	42 days
Pentavalent 2	70 days
Pentavalent 3	98 days
Oral polio vaccine (OPV) 1	42 days
Oral polio vaccine (OPV) 2	70 days
Oral polio vaccine (OPV) 3	98 days
Measles	273 days

### Ethics

This research is based on publicly available datasets from the DHS repository removed from any identifiable information. Also, because the data were completely anonymous, the authors did not need to seek additional ethical approval before using them. However, the authors complete the registration process and permission was granted to download and use the datasets.

### Statistical analysis

The trend is illustrated by the line graph over the period of study. The bivariate associations were investigated through the Chi-square test. The modeling was done using the logistic regression model (LRM) to estimate the association’s strength and the contribution of the independent variables to the variation of the dependent variable by estimating the odds ratios (95% confidence interval) and the coefficient of the model, respectively. We run four LRM for BCG, DPT, Polio, and Measles separately. The data processing and analyses were done using MS-Excel, STATA version 14, and IBM SPSS v25.

The logistic regression model can be expressed as,

$$\Pr \left({\mathrm{Y}}_i=1\right)=\frac{\exp\;\left({\mathrm{X}}_i\beta \right)}{1+\exp\;\left({\mathrm{X}}_i\beta \right)}$$

where, *Y*_*i*_ is a binary variable that takes a value of ‘1’ if the respondent received the vaccination and ‘0’ otherwise, X_*i*_ is a vector of independent variables and *β* is a vector of unknown parameters which contains the intercept parameter and the regression parameters associated with a set of covariates used in the study.

The fitted form of the model can be defined as,

$$\ln \left[\frac{\hat{P}}{1-{\hat{P}}_i}\right]={\hat{\beta}}_0+{\hat{\beta}}_1{X}_1+\dots +{\hat{\beta}}_k{X}_k$$

where, ${\hat{\beta}}_l\ \left(l=0,1,2,\dots, k\right)$ represents the estimated regression coefficient of the *l*^*th*^ independent variable in the study [42].

The *p*-value of the Chi-square test for measuring association among different characteristics and vaccination received by children aged 06–59 months are presented in Fig. 2. Variables significant at least once are included in the logistic regression models as covariates.

## Results

The proportion of various implemented vaccination by survey years are presented in Table 2. The results revealed that children’s rate of BCG vaccinations had been increased from 86.0 to 97.1% from 1994 to 2014. Also, the rate of DPT vaccination coverage increased about 35% from 1994 to 2014. The rate of Polio vaccination coverage was 68.9% in 1994 and reached 93.0% in 2014, and findings depict that the prevalence of Measles vaccination coverage is increased from 72.7 to 88% over the study period. About 24 percentage point has been increased for a total dose of DPT and Polio over the study period. The highest vaccination coverage rate has been observed for BCG (97.2%) and subsequently Polio (93.4%), DPT (93.1%), and Measles (89.7%) in 2011 (Table 2).

**Table 2:** Trend of receiving specific vaccination by children in Bangladesh, BDHS-1992 to BDHS-2014

Received vaccination	1993–94		1996–97		1999–2000		2004		2007		2011		2014	
	%	No. of Child	%	No. of Child	%	No. of Child	%	No. of Child	%	No. of Child	%	No. of Child	%	No. of Child
BCG	86.0	2058	88.1	3940	90.2	4618	93.6	4887	96.1	4433	97.2	6525	97.1	6064
DPT 3	68.0	1623	74.6	3331	72.0	3684	82.9	4328	89.5	4129	93.1	6249	92.4	5773
POLIO 3	68.9	1645	64.8	2894	71.7	3659	85.0	4437	90.4	4171	93.4	6270	93.0	5807
MEASLES	72.7	1733	76.8	3416	73.8	3778	80.1	4180	84.7	3909	89.7	6025	88.0	5484

**Fig. 1:**
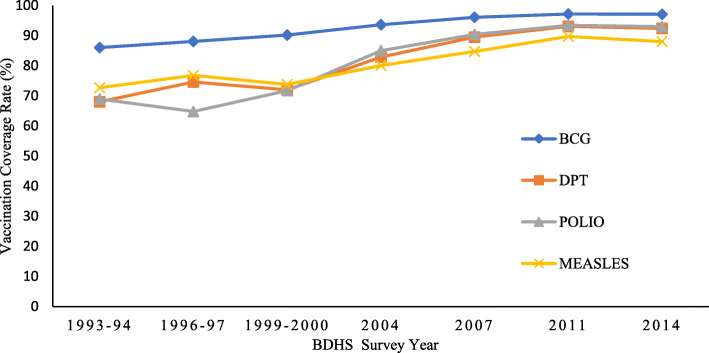
Trend of various vaccination received by children aged 12–59 months in Bangladesh

The BCG vaccination coverage was relatively high compared to other vaccines. Therefore, it is seen that the prevalence of BCG vaccination is increasing gradually over the study period. However, a similar pattern is observed for DPT and Measles vaccines. After 1996–97, the rate is dropped down slightly. However, after the year 2000, we observed an increasing trend for DPT and Measles vaccinations. In the case of Polio, the lowest percentage of receiving the vaccine is observed in 1996–97, and since then, a gradual upward trend is observed for the remaining survey years (Fig. 1).

**Fig. 2:**
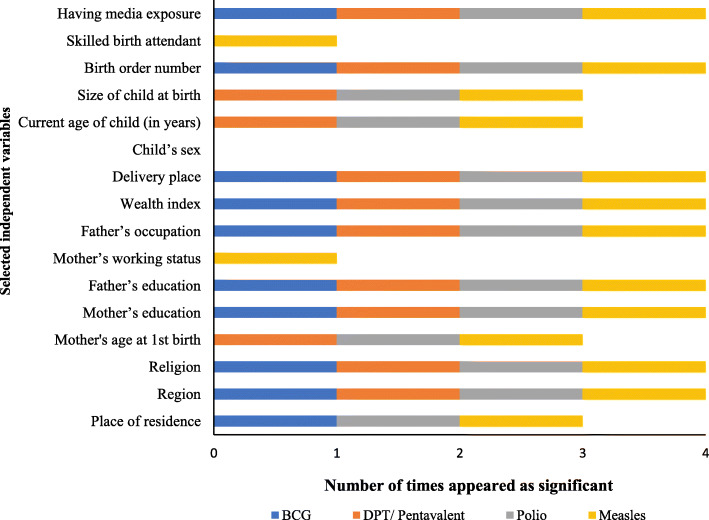
Number of times appeared significantly (*p* < 0.05) associated independent variables with various vaccinations, BDHS-2014

Results presented in Supplementary Table 1 disclosed the per cent distribution of various vaccination in light of different influential socioeconomic and demographic factors. Though there has been an upward trend for implementing various vaccination, however, the discrepancy is observed in urban and rural settings over the study period. Moreover, it has been found that in rural areas, 3.9 percentage points for BCG and polio, 2.3 percentage points for DPT/ Pentavalent, 4.1 percentage points for measles have been more increased than urban areas. Figure 2 presents the frequency of significant variables identified by the Chi-square test between variables from existing literature review and various vaccination coverage on the children aged 06–59 months. A detailed description of results for each vaccination coverage is mention in the subsequent sections.

### BCG vaccination

The findings depict that the urban/rural differentials in BCG vaccination coverage decreased from 5.1 percentage points in 1994 to 1.2 percentage points in 2014 (Supplementary Table 1), and there is a significant difference in the unadjusted odds ratio (OR) 1.45 (CI: 1.05–2.02) (Table 3). All regions except the Sylhet have the same situation for BCG vaccination in 2014. The highest (16.5%) percentage point of the prevalence of BCG vaccination coverage has been increased in the Chittagong region and lowest (3.5%) percentage point in the Barisal region (Supplementary Table 1). Although coverage variations among different regions have been decreased, there is still a significant difference in vaccination rates in the Sylhet region compared to other areas. The difference in BCG vaccination rates between Muslim and non-Muslim children decreased from 9.2% in 1994 to 1.6% in 2014. Also, there is a difference with OR 2.47 (CI: 1.15–5.30) in 2014 among Muslim and non-Muslim children (Table 3). The highest proportion of children have been BCG immunized whose mothers’ age at first birth is 25–29 years (Supplementary Table 1).

**Table 3:** Results of logistic regression for BCG, DPT, Polio, and Measles, BDHS-2014

Characteristics	BCG		DPT/ Pentavalent dose 3		Polio dose 3		Measles	
	Crude OR (CI)	Adjusted OR (CI)	Crude OR (CI)	Adjusted OR (CI)	Crude OR (CI)	Adjusted OR (CI)	Crude OR (CI)	Adjusted OR (CI)
**Place of residence**								
Urban	1.45 (1.05, 2.02)*	0.80 (0.45, 1.40)	–	–	1.26 (1.01, 1.56) *	0.64 (0.45, 0.90)*	1.24 (1.05, 1.46)*	0.76 (0.58, 1.0)
Rural	Ref	Ref	–	–	Ref	Ref	Ref	Ref
**Region**								
Barisal	Ref	Ref	Ref	Ref	Ref	Ref	Ref	Ref
Chittagong	0.82 (0.47, 1.43)	0.68 (0.27, 1.69)	0.85 (0.59, 1.22)	0.78 (0.46, 1.32)	0.93 (0.63, 1.36)	0.89 (0.52, 1.5)	1.08 (0.81, 1.44)	0.93 (0.61, 1.42)
Dhaka	1.57 (0.82, 2.97)	1.07 (0.38, 2.98)	1.37 (0.91, 2.05)	1.10 (0.62, 1.94)	1.41 (0.93, 2.15)	1.15 (0.65, 2.03)	1.34 (0.99, 1.83)	1.14 (0.73, 1.78)
Khulna	1.20 (0.61, 2.39)	1.27 (0.36, 4.50)	0.99 (0.64, 1.51)	0.73 (0.40, 1.32)	1.01 (0.65, 1.57)	0.87 (0.47, 1.59)	1.23 (0.87, 1.73)	0.86 (0.54, 1.38)
Rajshahi	1.58 (0.77, 3.23)	1.41 (0.44, 4.60)	1.08 (0.71, 1.64)	1.84 (0.78, 2.83)	0.98 (0.64,1.5)	1.45 (0.77, 2.72)	1.14 (0.83, 1.58)	1.23 (0.76, 1.99)
Rangpur	3.50 (1.38, 8.81)**	1.0 (empty)	1.71 (1.06, 2.74)*	4.44 (1.87, 10.57)**	1.97 (1.19, 3.25)**	4.55 (1.92, 10.8)**	1.81 (1.26, 2.59)**	2.04 (1.21, 3.46)**
Sylhet	0.30 (0.18, 0.50)***	0.22 (0.09, 0.50)***	0.34 (0.24, 0.48)***	0.34 (0.21, 0.56)***	0.35 (0.25, 0.49)***	0.37 (0.22, 0.6)***	0.43 (0.33, 0.56)***	0.34 (0.22, 0.51)
**Religion**								
Muslim	Ref	Ref	Ref	Ref	Ref	Ref	Ref	Ref
Others	2.47 (1.15, 5.30)*	1.68 (0.57, 4.94)	1.62 (1.08, 2.43)*	1.36 (0.76, 2.45)	1.63 (1.07, 2.48)*	1.44 (0.79, 2.64)	1.83 (1.30, 2.57)***	2.03 (1.23, 3.36)**
**Mother’s age 1st birth (years)**								
< 18	–	–	Ref	Ref	Ref	Ref	Ref	Ref
18–24	–	–	1.33 (1.1, 1.61)**	1.32 (0.99, 1.76)	1.34 (1.1, 1.63)**	1.28 (0.95, 1.72)	1.46 (1.25, 1.7)***	1.14 (0.91, 1.44)
25–29	–	–	1.48 (0.86, 2.54)	1.60 (0.61, 4.22)	1.46 (0.84, 2.55)	1.59 (0.6, 4.19)	1.63 (1.05, 2.54)*	1.23 (0.62, 2.49)
30 and above	–	–	5.43 (0.74, 39.42)	1 (empty)	1.61 (0.50, 5.20)	1 (empty)	1.76 (0.70, 4.44)	1 (empty)
**Mother’s education**								
No education	Ref	Ref	Ref	Ref	Ref	Ref	Ref	Ref
Primary	1.95 (1.40, 2.73)***	1.42 (0.81, 2.49)	1.7 (1.35, 2.15)***	1.88 (1.3, 2.72)**	1.58 (1.24, 2.0)***	1.73 (1.18, 2.51)**	1.47 (1.21, 1.79)***	1.59 (1.16, 2.16)**
Secondary	4.77 (3.29, 6.90)***	2.0 (0.96, 4.17)	3.5 (2.75, 4.45)***	2.42 (1.55, 3.76)***	3.55 (2.76, 4.56)***	2.26 (1.43, 3.56)***	3.34 (2.73, 4.08)***	2.17 (1.52, 3.11)***
Higher	10.21 (4.10, 25. 37)***	4.31 (0.46, 40.3)	9.6 (5.28, 17.42)***	6.51 (2.03, 20.89)**	7.33 (4.19, 12.83)***	3.27 (1.23, 8.72)*	7.46 (4.83, 11.51)***	4.62 (2.09, 10.25)***
**Husband’s education**								
No education	Ref	Ref	Ref	Ref	Ref	Ref	Ref	Ref
Primary	2.43 (1.72, 3.41)***	2.05 (1.13, 3.69)	1.64 (1.32, 2.04)***	1.35 (0.95, 1.91)	1.59 (1.27, 1.99)***	1.34 (0.94, 1.92)	1.53 (1.27, 1.83)***	1.23 (0.92, 1.64)
Secondary	3.50 (2.37, 5.16)***	1.27 (0.64, 2.52)	3.09 (2.38, 4.0)***	1.29 (0.84, 1.98)	3.33 (2.53, 4.39)***	1.39 (0.89, 2.17)	2.77 (2.25, 3.41)***	1.13 (0.81, (1.59)
Higher	8.71 (4.03, 18.80)***	2.47 (0.53, 11.5)	5.81 (3.75, 9.0)***	1.79 (0.77, 4.15)	5.11 (3.32, 7.87)***	1.54 (0.68, 3.46)	5.15 (3.68, 7.21)***	1.49 (0.8, 2.77)
**Mother’s working status**								
Non-employment	–	–	–	–	–	–	1.21 (1.03, 1.43)*	1.16 (0.9, 1.51)
Employment	–	–	–	–	–	–	Ref	Ref
**Father’s occupation**								
Farming	Ref	Ref	Ref	Ref	Ref	Ref	Ref	Ref
Non-farming	1.31 (0.91, 1.84)	0.72 (0.4, 1.28)	1.22 (0.98, 1.52)	0.94 (0.67, 1.31)	1.22 (0.97, 1.53)	0.92 (0.64, 1.32)	1.15 (0.96, 1.38)	0.99 (0.75, 1.31)
Professionals	3.27 (1.30, 8.20)**	0.36 (0.06, 2.16)	3.7 (1.98, 6.9)***	0.77 (0.26, 2.28)	3.41 (1.83, 6.38)***	0.94 (0.32, 2.78)	3.97 (2.39, 6.58)***	0.96 (0.42, 2.19)
Business	1.45 (0.97, 2.19)	0.56 (0.28, 1.10)	1.43 (1.09, 1.87)*	0.94 (0.62, 1.44)	1.43 (1.08, 1.89)*	0.91 (0.59, 1.41)	1.51 (1.21, 1.89)***	0.98 (0.7, 1.38)
Unemployed	1.31 (0.17, 9.76)	1 (empty)	0.75 (0.26, 2.16)	0.60 (0.12, 3.01)	0.69 (0.24, 2.0)	0.50 (0.1, 2.5)	0.97 (0.37, 2.55)	0.63 (0.16, 2.49)
Others	0.50 (0.25, 1.05)	0.39 (0.13, 1.21)	1.03 (0.54, 1.96)	1.0 (0.39, 2.56)	1.19 (0.59, 2.39)	1.13 (0.41, 3.12)	1.55 (0.84, 2.88)	1.53 (0.65, 3.64)
**Wealth index**								
Poorest	Ref	Ref	Ref	Ref	Ref	Ref	Ref	Ref
Poorer	1.49 (1.01, 2.17)*	0.84 (0.47, 1.52)	1.69 (1.31, 2.18)***	1.56 (1.06, 2.32)*	1.87 (1.43, 2.45)***	1.79 (1.19, 2.69)**	1.62 (1.31, 2.0)***	1.4 (1.02, 1.92)*
Middle	1.86 (1.25, 2.77)**	0.88 (0.44, 1.76)	2.26 (1.71, 2.97)***	1.45 (0.94, 2.25)	2.51 (1.88, 3.34)***	1.7 (1.08, 2.67)*	2.12 (1.69, 2.66)***	1.45 (1.01, 2.06)*
Richer	2.36 (1.54, 3.60)***	0.88 (0.39, 1.98)	2.33 (1.77, 3.05)***	1.25 (0.76, 2.06)	2.54 (1.91, 3.37)***	1.55 (0.93, 2.61)	2.19 (1.75, 2.73)***	1.61 (1.07, 2.44)*
Richest	7.89 (3.94, 15.80)***	1.82 (0.50, 6.66)	5.18 (3.6, 7.47)***	2.13 (1.07, 4.26)*	5.2 (3.6, 7.53)***	2.73 (1.33, 5.6)**	3.98 (3.03, 5.22)***	2.60 (1.5, 4.5)**
**Delivery place**								
At home	Ref	Ref	Ref	Ref	Ref	Ref	Ref	Ref
At health facility	4.53 (2.4, 8.55)***	1.74 (0.86, 3.52)	2.25 (1.66, 3.06)***	1.08 (0.76, 1.55)	2.22 (1.63, 3.04)***	1.13 (0.78, 1.63)	1.9 (1.51, 2.4)***	0.94 (0.71, 1.25)
**Current age of child (in years)**								
≤ 1	–	–	Ref	Ref	Ref	Ref	Ref	Ref
2	–	–	1.42 (1.1, 1.85)**	1.66 (1.26, 2.2)***	1.43 (1.1, 1.87)**	1.68 (1.26, 2.25)***	1.38 (1.12, 1.7)**	1.56 (1.25, 1.95)***
3	–	–	1.13 (0.88, 1.44)	–	1.24 (0.96, 1.61)	–	1.35 (1.09, 1.66)**	–
4	–	–	1.40 (1.07, 1.82)*	–	1.42 (1.09, 1.86)*	**–**	1.4 (1.14, 1.73)**	**–**
**Size of child at birth**								
Very large	–	–	1.55 (0.6, 3.98)	0.93 (0.34, 2.51)	1.47 (0.57, 3.79)	0.83 (0.30, 2.25)	1.68 (0.74, 3.84)	1.08 (0.46, 2.55)
Larger thanaverage	–	–	1.12 (0.63, 1.99)	0.79 (0.43, 1.47)	1.03 (0.58, 1.83)	0.72 (0.38, 1.35)	1.09 (0.67, 1.78)	0.86 (0.51, 1.46)
Average	–	–	1.68 (1.05, 2.7)*	1.23 (0.73, 2.05)	1.65 (1.02, 2.68)*	1.19 (0.71, 2.02)	1.31 (0.88, 1.97)	1.0 (0.65, 1.55)
Smaller than average	–	–	0.99 (0.57, 1.70)	0.97 (0.54, 1.73)	1.1 (0.63, 1.94)	1.08 (0.59, 1.98)	0.85 (0.54, 1.35)	0.82 (0.5, 1.34)
Very small	–	–	Ref	Ref	Ref	Ref	Ref	Ref
**Birth order number**								
1	3.72 (2.52, 5.51)***	2.38 (1.18, 4.82)*	2.33 (1.82, 2.99)***	0.96 (0.64, 1.45)	2.4 (1.86, 3.11)***	1.0 (0.66, 1.52)	2.35 (1.91, 2.89)***	1.1 (0.78, 1.54)
2	2.56 (1.75, 3.74)***	1.61 (0.81, 3.22)	2.02 (1.82, 2.99)***	1.33 (0.86, 2.05)	2.15 (1.64, 2.8)***	1.47 (0.94, 2.3)	1.99 (1.61, 2.46)***	1.1 (0.78, 1.56)
3	1.64 (1.09, 2.45)**	0.67 (0.37, 1.21)	1.67 (1.24, 2.23)**	0.96 (0.62, 1.47)	1.65 (1.22, 2.22)**	0.98 (0.63, 1.53)	1.67 (1.31, 2.13)***	0.96 (0.67, 1.37)
4 and above	Ref	Ref	Ref	Ref	Ref	Ref	Ref	Ref
**Skilled birth attendant**								
No	–	–	–	–	–	–	1.37 (1.01, 1.85)*	1.27 (0.91, 1.76)
Yes	–	–	–	–	–	–	Ref	Ref
**Having media exposure**								
No	Ref	Ref	Ref	Ref	Ref	Ref	Ref	Ref
Yes	3.20 (2.22, 4.60)***	2.39 (1.17, 4.90)	2.35 (1.89, 2.91)***	1.49 (1.0, 2.23)*	2.37 (1.9, 2.97)***	1.46 (0.97, 2.2)	2.13 (1.8, 2.51)***	1.2 (0.88, 1.63)

*‘*p* < 0.05’, **‘*p* < 0.01’, ***‘*p* < 0.001’; *Ref* Reference category; *OR* Odds ratio; *CI* 95% confidence interval

The proportion of vaccination coverage during the study years has been increased with the increases of mothers’ or/and fathers’ education levels. An increment is observed in BCG vaccination coverage from illiterate mothers to higher educated mothers (Supplementary Table 1). Children of higher educated mothers have ten times (OR 10.21; CI: 4.10–25.37) more chance to be immunized compared to the children of illiterate mothers. Also, the likelihood of receiving the BCG vaccine is eight times (OR 8.71; CI: 4.03–18.80) more whose father is higher educated compared to illiterate father (Table 3). There exists an almost similar condition of vaccination implementation for employed and non-employed mothers. The child’s sex and current age, size of child at birth, and skilled birth attendant do not favor making the difference of BCG vaccination execution rate. Moreover, the BCG vaccination has been less implemented on the children whose fathers are farmers rather than other occupations. Children born in a household with higher socio-economic status had a significantly higher chance of being BCG immunized than children in a poorer household. Delivery at health facilities and having media exposure have shown the more vaccine implementation rate over the study period (Supplementary Table 1). The children have a chance of being 4.5 times (OR 4.53; CI: 2.4–8.55) more BCG vaccinated if they were born at health facilities and of being 3.2 times (OR 3.20; CI: 2.22–4.60) more if their mothers’ have media exposure compared to their counterparts.

### DPT/ pentavalent vaccination

This study results disclosed that the proportion of DPT vaccinated children increased 72.7 to 94.6% from 1994 to 2014 in urban areas while from 67.5 to 91.7% from 1994 to 2014 in rural areas, but urban/rural differentials are not significant (Supplementary Table 1). The current vaccination coverage status of all regions except the Sylhet division has gradually increased over the study period. However, there is significantly lower coverage of DPT vaccination in Sylhet than in the other regions of Bangladesh. It has been seen that the vaccine coverage on Muslim children is lower than the non-Muslim children from 1994 to 2011, but an equal rate of coverage has been observed in the recent survey. Also, the DPT vaccine implementation rate is higher with the increases of mothers’ age at the 1st birth. Although the proportion of DPT immunized children with illiterate mothers increased about 25% from 1994 to 2014 (Supplementary Table 1). Still, there is a significant difference between children with primary (OR 1.7; CI: 1.35–2.15), secondary (OR 3.5; CI: 2.75–4.45), and higher (OR 9.6; CI: 5.28–17.42) educated mothers. Similarly, the vaccination coverage rate of children with illiterate fathers is significantly different (OR 5.81; CI: 3.75–9.0) from children with higher educated fathers (Table 3).

The proportion of vaccine implementation is more for non-employed mothers than employed mothers, but the difference is not statistically significant. The DPT vaccination has been more implemented by 3.7 times (OR 3.7; CI: 1.98–6.9) on children whose fathers are professionals than children whose fathers are farmers. Children who lived in a household with a better wealth index have a chance of being more DPT vaccinated than that of poorer households. Vaccination coverage rate has been different between children who were born at health facility and born at home (*p* < 0.001); and mother has media exposure have shown the more (OR 2.35; CI: 1.89–2.91) vaccine implementation rate during the study period (Table 3). No significant pattern (increased or decreased in implementation) was exhibited with the child’s current age and skilled birth attendant. DPT vaccination coverage is significantly decreased with higher birth order (Supplementary Table 1).

### Polio vaccination

Table 3 revealed that the Polio vaccine coverage differentials in urban/rural context decreased from 6.4% in 1994 to 2.5% in 2014 (Supplementary Table 1). There is a statistically significant difference between urban and rural areas in Polio vaccination coverage (OR 1.26; CI: 1.01–1.56). The highest proportion and rate of change of Polio vaccine implementation exist in the Dhaka region rather than the other regions. The lowest execution of the Polio vaccine is observed in the Sylhet region with an odds ratio of 0.35 (CI: 0.25–0.49) over the study period. The proportion of children have been vaccinated from the non-Muslim community is 63% (OR 1.63; CI: 1.07–2.48) higher than their Muslim counterpart (Table 3); though the disparity is decreased 10.4 percentage point in 1994 to 0.9 percentage point in 2014 (Supplementary Table 1). Moreover, the Polio vaccination rate is increased with the increases of mothers’ age at the 1st birth and demonstrated a significant difference between adolescent mothers and mothers aged 18–24 years at first birth with an odds ratio of 1.34 (CI: 1.24–2.0) (Table 3).

Children with educated parents have a chance of being more Polio immunized compared to the parents with no formal education, and this vaccination possibility is more extensive for higher educated mothers than higher educated fathers with an odds ratio of 7.33 (CI: 4.19–12.83) and 5.11 (CI: 3.32–7.87) respectively. The percentage of polio vaccine implementation is more for non-employed mothers than employed mothers and does not reveal any statistical difference. The proportion of children whose father is a professional has been vaccinated 3.4-fold (CI: 1.83–6.38) more than the children whose father is a farmer (Table 3).

The children born in a household with upper wealth quintiles have significantly more chances of being Polio vaccinated than those with lower wealth quintiles. The proportion (OR 2.2; CI: 1.63–3.04) of vaccination and increasing rate, from 70.6% in 1994 to 96.2% in 2014, of the Polio vaccine, is more for health facility delivery than delivery at home. Furthermore, Polio vaccination is significantly decreased with increases in the birth order number. Although the differentials between having and not having media exposure are decreased over the study period (Supplementary Table 1), children whose mother’s having media exposure have been more Polio immunized with an odds ratio of 2.37 (CI: 1.9–2.97) compared to children whose mothers having no media exposure (Table 3).

### Measles vaccination

It has been found that there is a significant difference in implementing the Measles vaccine between urban and rural areas with an odds ratio of 1.24 (CI: 1.05–1.46) (Table 3) though the discrepancy is decreased from 7.2% in 1994 to 3.1% in 2014 (Supplementary Table 1). The highest proportion of measles vaccination coverage is in the Khulna region, and the lowest is in the Sylhet region. Also, the lowest increase, 64.9% in 1997 to 74% in 2014 of Measles vaccination coverage, occurred in the Sylhet region over the study period compared to the other regions. Measles vaccination coverage is 46% lower in the Muslim community than their counterparts, but the gap decreased from 13.7% in 1994 to 3.2% in 2014. The highest percentage of vaccination implemented is observed among children whose mothers’ age at first birth is 25–29 years over the study time (Supplementary Table 1). Moreover, the vaccination rate is significantly different from children whose mothers were adolescent at their first birth (Table 3).

The Measles vaccination rate of children increased from 65.1 to 79.8% for illiterate mothers and 66.6 to 80.9% for illiterate fathers from 1994 to 2014 (Supplementary Table 1). Also, there is a significant difference among the children with educated parents, and the rate is increased with increasing the parents’ education level. Interestingly, the proportion of measles vaccine implementation is higher (OR 1.21; CI: 1.03–1.43) on the children whose mothers are unemployed and mothers with unskilled birth attendants (OR 1.37; CI: 1.01–1.85). Moreover, children have more chances of being more Measles immunized whose fathers are professional (OR 3.97; CI: 2.39–6.58) and businessmen (OR 1.51; CI 1.21–1.89) than those with fathers involved with farming. Improved socio-economic status leads to a significant increase in the Measles vaccination coverage rate. Having media exposure (OR 2.13; CI: 1.8–2.51; ref.: having no media exposure) and health facility delivery (OR 1.9; CI: 1.51–2.4; ref.: delivery at home) play a significant role to increase the Measles vaccination coverage in 2014 (Table 3). The percentage of children with measles vaccine immunized is decreasing with the increment of the birth order number.

## Discussion

The vaccine-preventable diseases like pneumonia, diarrhoea, and measles collectively responsible for half of the deaths recorded in 2013 [13]. That is, complete immunization coverage may prevent 50% of illness and death in developing countries. This study exposes the trend and determining factors of the four vaccinations coverage from 1992 to 2014 in Bangladesh. The logistic regression analysis results revealed that several influencing factors, including administrative region, mother’s education, socio-economic status, the current age of children, and having media exposure, have been significant for BCG, DPT/Pentavalent 3, Polio, and Measles vaccine implementation respectively.

This study has revealed that the complete dose of DPT has been increased by about 24 percentage point, from 1994 to 2014 and Polio vaccinations received by children aged 06–59 months were increased from 68.9 to 93.0% over the period 1994 to 2014. The rate of BCG vaccinations has been increased from 86.0% in 1994 to 97.1% in 2014 and it is increased from 72.7 to 88% from 1994 to 2014 for measles vaccination. The highest vaccination coverage rate has been observed for BCG and the lowest for Measles vaccination over the period 2000 to 2014 (Fig. 1). The reason might be that the recommended age period of children for BCG to Measles vaccination is increasing while mothers or caregivers have been less concerned about immunization with increasing the children’s age and its side effects [43].

The adjusted odds ratio demonstrated that the children born in the Sylhet division were less likely to receive vaccines, whereas children born in Rangpur/Rajshahi division were more likely to receive vaccines. This scenario is observed for all kinds of vaccination coverage received by children. Similar findings are also observed by other researchers [3, 29, 33, 44]. Moreover, Sheikh et al. (2018) highlighted that the Sylhet division primarily covers a remote hilly and riverine area, and the communication system is more fragile than other regions of the country [33]. The mother’s education level has been significantly associated with DPT, Polio, and Measles vaccination coverage. Children whose mothers had completed a primary, secondary, and a higher level of education have been 1.88, 2.42, and 6.51 times more likely to receive DPT vaccine; have been 1.73, 2.26, and 3.27 times more likely to receive Polio vaccine; 1.59, 2.17, and 4.62 times more likely to receive Measles vaccine respectively as compared with children whose mothers had no formal education. The significance of a mother’s education has also been found in many existing papers of home and abroad [2, 3, 28–30, 32, 33, 44–46]. However, such association has not been observed in scenarios of BCG vaccination though a positive relationship has been seen in the unadjusted odds ratio.

This study demonstrated that children born in households with higher wealth quintiles have significantly higher chances of being immunized against DPT, Polio, and Measles than children born in the poorest wealth quintile households. Similar results have been found in other settings [3, 28, 29, 33, 44–46]. Moreover, no significant association is observed for BCG vaccination after adjusting other variables in the model. Children’s current age is determined as another highly significant factor in receiving DPT, Polio, and Measles vaccination. It is observed that the children aged 24 months have 1.66, 1.68, and 1.56 times more likely to immunize against DPT, Polio, and Measles vaccination, respectively, compared to children aged 12 months. A positive association between vaccination coverage and children’s current age has been seen previously [2, 28, 44]. Our research has also revealed that, after adjusting other variables to the model, lower birth order (AOR 2.38; CI: 1.18, 4.82) is found as a significant factor to have more likely of receiving BCG vaccination compared to higher birth order (≥4) and this result is similar with others [28, 44]. Children who lived in a household having media exposure have 1.5 times (AOR 1.49; CI: 1.0, 2.23) more likely to be immunized against DPT, and these findings are supported by the others [3, 44, 45]. The place of residence is a significant factor for receiving Polio vaccination, and the likelihood of receiving the measles vaccine is twice (AOR 2.03; CI: 1.23, 3.36) for non-Muslim compared to their Muslim counterpart. Although the mother’s working status has been found insignificantly but interestingly, this contradicts to the findings of other settings that children of unemployed mothers failed to receive timely vaccinations for BCG/measles [33].

### Strength and limitation

The strength of the study is that it is using seven nationally representative demographic and health survey data. Therefore, the authors believe that the findings are representative of a whole nation. Additionally, in this study, we consider four vaccination coverage. Despite these strengths, the inclusion criteria are children aged 06–59 months. The future study should focus on expanding the analysis to include other explanatory variables such as geospatial attributes related to child health and using the up-to-date data.

## Conclusion

This study explores a trend and pattern of four, namely BCG, Diphtheria, Pertussis, Tetanus (DPTs)/Pentavalents dose 3, Polio dose 3, and Measles vaccination coverage from 1993 to 2014 using Bangladesh demographic health survey data. Some significant determinants have also been found for the vaccination coverage distinctly of children age 06–59 months using the BDHS 2014 data. During the study period, coverage trends showed that the rate of measles vaccine implementation at the 273rd day of a child’s age is lower among all analyzed vaccination. The findings of this paper revealed that the administrative region is significantly associated with implementing all four-vaccination coverage. Moreover, being an educated mother’s child, better socio-economic condition, and comparatively older children put some extra advantage of being immunized against DPT, Polio, and Measles diseases. Birth order numbers for BCGs, having media exposure for DPTs, place of residence for Polio, religion for Measles have been found as statistically associated determinants. Finally, these findings recommend that mother’s or caregiver’s counselling procedures at the time of postnatal care have to continue until children reach their second birthday, and concern officials have to pay more concentration on discussed significant factors, especially in the Sylhet region.

## Supplementary Information

**Additional file 1.**

## Data Availability

After registration, the data set is available via the following access link http://dhsprogram.com/data/available-datasets.cfm.
