# Supplementary material for: Trends and determinants of vaccination among children aged 06–59 months in Bangladesh: country representative survey from 1993 to 2014
Source: BMC Public Health. 2021 Aug 21;21:1578. doi: 10.1186/s12889-021-11576-0 (PMC8379560; doi:10.1186/s12889-021-11576-0)
Supplement: Supplementary file 1 — Additional file 1. [file 12889_2021_11576_MOESM1_ESM.docx]

**Supplementary Table 1:** Percentage distribution of children aged 06-59 months who received specific vaccination in Bangladesh, BDHS 1992 to BDHS 2014

| Characteristics | BCG | | | | | | | DTP/ Pentavalent | | | | | | | Polio | | | | | | | Measles | | | | | | |
| --- | --- | --- | --- | --- | --- | --- | --- | --- | --- | --- | --- | --- | --- | --- | --- | --- | --- | --- | --- | --- | --- | --- | --- | --- | --- | --- | --- | --- |
|  | 1993-94 | 1996-97 | 1999-2000 | 2004 | 2007 | 2011 | 2014 | 1993-94 | 1996-97 | 1999-00 | 2004 | 2007 | 2011 | 2014 | 1993-94 | 1996-97 | 1999-00 | 2004 | 2007 | 2011 | 2014 | 1993-94 | 1996-97 | 1999-00 | 2004 | 2007 | 2011 | 2014 |
| **Place of residence** | | | | | | | | | | | | | | | | | | | | | | | | | | | | |
| Urban | 90.6 | 94.1 | 92.6 | 94.5 | 97.0 | 98.6 | 98.0 | 72.7 | 81.9 | 80.8 | 86.1 | 91.6 | 95.3 | 94.6 | 74.7 | 69.0 | 79.5 | 87.7 | 92.0 | 95.2 | 94.9 | 79.2 | 86.8 | 80.6 | 81.9 | 86.2 | 90.7 | 90.3 |
| Rural | 85.5 | 87.6 | 89.8 | 93.4 | 95.8 | 96.8 | 96.8 | 67.5 | 73.9 | 70.2 | 82.1 | 88.9 | 92.4 | 91.7 | 68.3 | 64.4 | 70.1 | 84.3 | 90.0 | 92.8 | 92.4 | 72.0 | 75.8 | 72.5 | 79.6 | 84.3 | 89.5 | 87.2 |
| **Region** |  |  |  |  |  |  |  |  |  |  |  |  |  |  |  |  |  |  |  |  |  |  |  |  |  |  |  |  |
| Barisal | 93.8 | 91.5 | 93.8 | 97.0 | 97.2 | 98.0 | 97.3 | 83.0 | 80.5 | 76.7 | 82.6 | 91.1 | 94.0 | 93.8 | 81.9 | 71.7 | 77.6 | 87.7 | 91.8 | 94.8 | 93.3 | 85.0 | 82.4 | 77.4 | 81.3 | 86.5 | 91.4 | 87.5 |
| Chittagong | 80.5 | 85.0 | 91.9 | 90.8 | 94.6 | 95.3 | 97.0 | 62.3 | 71.4 | 75.7 | 80.7 | 87.2 | 89.5 | 91.2 | 63.3 | 61.0 | 77.4 | 82.7 | 87.7 | 90.4 | 92.7 | 67.4 | 71.5 | 78.9 | 78.0 | 81.8 | 87.7 | 88.3 |
| Dhaka | 82.4 | 85.3 | 88.2 | 94.5 | 96.3 | 97.8 | 97.6 | 59.4 | 70.7 | 69.9 | 82.5 | 88.6 | 93.8 | 94.3 | 59.7 | 61.5 | 70.6 | 85.3 | 90.5 | 93.9 | 94.8 | 65.7 | 73.2 | 69.4 | 79.4 | 82.7 | 88.8 | 89.9 |
| Khulna | 93.9 | 96.6 | 94.3 | 97.5 | 99.0 | 99.3 | 97.9 | 85.1 | 86.9 | 80.3 | 90.4 | 96.1 | 96.8 | 94.1 | 87.0 | 73.5 | 77.8 | 92.4 | 96.6 | 97.2 | 94.4 | 85.5 | 91.3 | 82.9 | 87.7 | 93.4 | 93.3 | 90.5 |
| Rajshahi | 92.2 | 92.9 | 91.7 | 94.6 | 97.6 | 97.3 | 98.0 | 74.3 | 78.6 | 70.4 | 86.2 | 92.6 | 94.4 | 92.7 | 76.0 | 69.9 | 66.8 | 86.6 | 93.4 | 94.4 | 92.4 | 79.1 | 82.4 | 73.2 | 82.2 | 89.4 | 91.9 | 87.5 |
| Rangpur |  |  |  |  |  | 98.8 | 99.4 |  |  |  |  |  | 96.7 | 96.7 |  |  |  |  |  | 96.3 | 97.0 |  |  |  |  |  | 94.1 | 93.1 |
| Sylhet |  | 80.1 | 79.1 | 87.6 | 91.3 | 94.4 | 91.8 |  | 65.7 | 57.4 | 71.8 | 82.1 | 88.3 | 81.9 |  | 57.0 | 58.9 | 74.2 | 81.8 | 88.9 | 83.3 |  | 64.9 | 62.2 | 72.0 | 76.9 | 84.3 | 74.0 |
| **Religion** | | | | | | | | | | | | | | | | | | | | | | | | | | | | |
| Muslim | 84.9 | 87.6 | 89.8 | 93.3 | 95.9 | 97.3 | 97.0 | 66.9 | 73.8 | 71.4 | 82.2 | 89.3 | 92.9 | 92.4 | 67.7 | 64.1 | 71.0 | 84.4 | 90.3 | 93.3 | 93.0 | 71.1 | 75.9 | 73.2 | 79.2 | 84.7 | 89.6 | 87.7 |
| Others | 94.1 | 94.2 | 94.1 | 98.1 | 98.1 | 96.2 | 98.6 | 76.5 | 83.3 | 77.2 | 91.2 | 91.7 | 94.6 | 92.3 | 78.1 | 72.9 | 77.3 | 92.9 | 91.8 | 94.7 | 93.9 | 84.8 | 86.4 | 79.1 | 90.8 | 85.1 | 91.6 | 90.9 |
| **Mother's age at 1st birth** | | | | | | | | | | | | | | | | | | | | | | | | | | | | |
| Less than 18 | 84.5 | 86.9 | 89.0 | 92.5 | 95.2 | 96.7 | 96.8 | 66.5 | 73.0 | 70.0 | 80.7 | 88.2 | 92.3 | 91.3 | 67.5 | 63.0 | 69.8 | 82.9 | 89.2 | 92.6 | 92.1 | 70.3 | 74.8 | 71.3 | 77.8 | 83.1 | 88.5 | 85.8 |
| 18-24 | 87.8 | 90.0 | 92.0 | 95.3 | 97.0 | 97.5 | 97.3 | 69.5 | 77.4 | 75.0 | 86.1 | 90.6 | 93.7 | 93.3 | 70.5 | 68.2 | 74.3 | 88.0 | 91.4 | 94.0 | 93.8 | 75.4 | 80.2 | 77.7 | 83.4 | 86.0 | 90.7 | 89.8 |
| 25-29 | 91.3 | 96.6 | 90.0 | 96.3 | 99.6 | 99.8 | 98.8 | 75.1 | 85.1 | 72.4 | 88.1 | 97.8 | 96.1 | 94.1 | 75.1 | 74.6 | 73.9 | 89.6 | 96.8 | 96.8 | 94.7 | 81.1 | 86.4 | 76.3 | 87.2 | 94.2 | 94.9 | 90.8 |
| 30 and above | 90.5 | 96.2 | 97.7 | 97.6 | 100.0 | 98.8 | 97.8 | 90.0 | 55.3 | 65.5 | 96.4 | 95.2 | 96.2 | 97.8 | 90.0 | 50.1 | 69.6 | 96.4 | 98.3 | 96.2 | 94.3 | 90.5 | 79.2 | 60.5 | 83.7 | 100.0 | 91.9 | 87.7 |
| **Mother's Education** | | | | | | | | | | | | | | | | | | | | | | | | | | | | |
| No education | 81.3 | 83.1 | 85.9 | 89.3 | 90.9 | 91.6 | 93.9 | 60.3 | 68.6 | 64.7 | 74.4 | 80.2 | 83.8 | 85.3 | 61.8 | 59.9 | 64.6 | 76.7 | 82.1 | 84.4 | 87.4 | 65.1 | 70.5 | 65.4 | 71.6 | 74.9 | 79.9 | 79.8 |
| Primary | 90.3 | 93.4 | 92.2 | 95.0 | 96.7 | 97.3 | 95.8 | 75.7 | 79.6 | 74.3 | 83.6 | 89.6 | 92.8 | 90.1 | 75.9 | 70.5 | 74.5 | 86.4 | 90.3 | 93.1 | 90.3 | 79.0 | 82.3 | 75.9 | 81.8 | 84.1 | 88.2 | 83.1 |
| Secondary | 95.3 | 97.4 | 96.4 | 97.5 | 99.0 | 99.5 | 98.6 | 81.9 | 86.4 | 82.9 | 92.5 | 95.4 | 96.9 | 95.3 | 81.7 | 73.8 | 81.5 | 93.7 | 95.7 | 97.1 | 95.8 | 88.0 | 89.2 | 87.0 | 88.5 | 91.7 | 94.4 | 92.3 |
| Higher | 98.9 | 99.7 | 98.8 | 99.5 | 100.0 | 99.8 | 99.2 | 92.0 | 96.9 | 91.0 | 98.0 | 97.9 | 99.2 | 98.5 | 92.0 | 72.0 | 88.7 | 98.7 | 99.3 | 99.1 | 97.8 | 98.9 | 99.0 | 97.1 | 95.2 | 94.2 | 98.5 | 96.3 |
| **Father's education** | | | | | | | | | | | | | | | | | | | | | | | | | | | | |
| No education | 82.2 | 83.4 | 85.8 | 90.6 | 93.2 | 94.3 | 94.1 | 62.5 | 69.1 | 65.5 | 76.8 | 83.4 | 87.6 | 87.1 | 63.7 | 60.0 | 66.3 | 79.2 | 85.3 | 88.4 | 88.3 | 66.6 | 70.4 | 66.6 | 72.9 | 77.3 | 84.3 | 80.9 |
| Primary | 86.5 | 89.9 | 92.1 | 94.5 | 96.3 | 97.5 | 97.6 | 68.5 | 75.6 | 72.3 | 83.5 | 90.1 | 93.7 | 91.8 | 69.2 | 66.7 | 72.7 | 85.7 | 90.5 | 93.7 | 92.4 | 73.2 | 78.3 | 72.8 | 81.1 | 85.5 | 89.8 | 87.4 |
| Secondary | 89.8 | 94.3 | 94.5 | 96.5 | 98.5 | 98.8 | 98.2 | 76.1 | 81.9 | 78.7 | 88.7 | 94.4 | 95.7 | 95.5 | 76.3 | 72.1 | 76.0 | 90.6 | 94.8 | 95.8 | 96.1 | 78.8 | 84.9 | 82.4 | 87.1 | 90.1 | 92.0 | 91.3 |
| Higher | 97.8 | 98.1 | 97.1 | 97.6 | 99.4 | 99.6 | 99.4 | 78.6 | 89.2 | 86.7 | 94.7 | 97.0 | 98.7 | 97.6 | 80.8 | 72.0 | 84.3 | 96.1 | 97.2 | 98.9 | 97.2 | 91.4 | 93.9 | 91.5 | 92.9 | 95.0 | 97.2 | 95.6 |
| **Mother’s working status** | | | | | | | | | | | | | | | | | | | | | | | | | | | | |
| No | 85.8 | 88.1 | 90.3 | 93.9 | 96.0 | 97.1 | 97.3 | 68.3 | 74.5 | 71.9 | 83.0 | 90.0 | 93.2 | 92.8 | 69.0 | 63.3 | 70.9 | 85.1 | 90.7 | 93.4 | 93.3 | 72.4 | 76.7 | 73.9 | 80.4 | 85.4 | 89.9 | 88.8 |
| Yes | 87.6 | 88.1 | 90.2 | 92.6 | 96.3 | 97.5 | 96.7 | 66.3 | 74.7 | 72.4 | 82.8 | 88.1 | 92.1 | 91.4 | 68.6 | 67.7 | 74.8 | 84.4 | 89.6 | 93.1 | 92.4 | 74.8 | 76.8 | 73.8 | 78.8 | 83.1 | 88.3 | 85.7 |
| **Father's occupation** | | | | | | | | | | | | | | | | | | | | | | | | | | | | |
| Farming | 85.2 | 86.7 | 89.3 | 93.7 | 96.2 | 95.4 | 95.2 | 65.4 | 72.0 | 68.2 | 82.2 | 88.1 | 90.1 | 89.1 | 66.3 | 64.1 | 66.7 | 84.0 | 89.5 | 90.7 | 89.9 | 70.4 | 74.6 | 68.4 | 79.1 | 84.0 | 86.8 | 83.9 |
| Non-farming | 86.4 | 88.5 | 89.5 | 93.1 | 95.2 | 97.6 | 97.7 | 68.1 | 74.0 | 70.9 | 81.5 | 88.2 | 93.4 | 92.9 | 69.2 | 63.6 | 70.8 | 83.6 | 89.0 | 93.7 | 93.6 | 72.6 | 76.1 | 74.0 | 78.4 | 82.1 | 89.4 | 87.6 |
| Professionals | 95.6 | 92.0 | 96.8 | 98.1 | 99.7 | 99.2 | 98.8 | 78.0 | 83.9 | 85.8 | 92.9 | 98.6 | 97.9 | 96.8 | 80.9 | 70.6 | 84.8 | 95.2 | 99.5 | 97.9 | 96.9 | 85.5 | 87.3 | 92.2 | 93.5 | 93.2 | 96.5 | 95.3 |
| Business | 85.0 | 89.7 | 91.0 | 94.3 | 96.9 | 98.3 | 97.6 | 69.9 | 78.3 | 74.4 | 85.3 | 92.0 | 95.5 | 94.2 | 70.5 | 67.1 | 74.7 | 87.9 | 92.6 | 95.6 | 94.5 | 74.0 | 79.9 | 76.0 | 83.0 | 88.8 | 93.0 | 91.0 |
| Unemployed | 90.2 | 94.6 | 86.3 | 97.3 | 95.6 | 96.6 | 99.0 | 83.2 | 87.8 | 79.6 | 91.4 | 91.4 | 93.6 | 93.7 | 83.2 | 61.6 | 75.8 | 89.7 | 90.6 | 92.8 | 93.7 | 83.2 | 74.3 | 79.5 | 81.1 | 88.0 | 89.9 | 92.7 |
| Others | 87.5 | 73.4 | 94.1 | 89.2 | 97.4 | 96.4 | 96.6 | 72.3 | 72.7 | 77.4 | 77.9 | 96.0 | 83.3 | 94.6 | 72.3 | 62.5 | 83.4 | 81.1 | 95.6 | 83.3 | 95.2 | 78.9 | 67.1 | 78.6 | 78.9 | 92.8 | 79.8 | 93.7 |
| **Wealth index** | | | | | | | | | | | | | | | | | | | | | | | | | | | | |
| Poorest |  |  |  | 88.7 | 94.3 | 93.6 | 95.1 |  |  |  | 72.6 | 86.0 | 87.8 | 86.0 |  |  |  | 75.0 | 87.2 | 87.9 | 86.8 |  |  |  | 69.1 | 79.9 | 83.3 | 79.2 |
| Poorer |  |  |  | 94.4 | 95.5 | 96.7 | 97.1 |  |  |  | 84.4 | 87.6 | 91.2 | 91.6 |  |  |  | 86.8 | 88.7 | 91.8 | 93.0 |  |  |  | 82.3 | 83.4 | 88.4 | 87.8 |
| Middle |  |  |  | 94.7 | 95.6 | 98.5 | 96.4 |  |  |  | 83.0 | 89.8 | 94.5 | 93.7 |  |  |  | 85.2 | 90.3 | 94.7 | 94.5 |  |  |  | 81.1 | 84.5 | 91.1 | 90.5 |
| Richer |  |  |  | 95.2 | 97.4 | 98.4 | 98.1 |  |  |  | 86.7 | 91.0 | 96.0 | 94.5 |  |  |  | 88.7 | 92.7 | 96.5 | 94.8 |  |  |  | 83.5 | 88.5 | 92.1 | 89.6 |
| Richest |  |  |  | 97.6 | 98.3 | 99.7 | 99.2 |  |  |  | 92.9 | 94.4 | 97.6 | 97.6 |  |  |  | 94.1 | 94.5 | 97.7 | 97.4 |  |  |  | 89.6 | 89.0 | 95.8 | 94.5 |
| **Delivery place** | | | | | | | | | | | | | | | | | | | | | | | | | | | | |
| Delivery at home | 86.0 | 87.1 | 88.3 | 92.2 | 94.8 | 96.4 | 96.8 | 67.4 | 73.2 | 69.8 | 80.3 | 87.1 | 91.5 | 90.1 | 68.4 | 63.7 | 69.7 | 82.5 | 88.1 | 92.0 | 90.9 | 71.9 | 75.0 | 71.4 | 78.2 | 82.9 | 88.2 | 84.2 |
| Delivery at health facility | 86.2 | 91.2 | 94.4 | 96.8 | 98.2 | 99.6 | 99.2 | 69.8 | 78.4 | 76.6 | 88.5 | 93.3 | 98.2 | 96.2 | 70.6 | 68.0 | 75.8 | 90.3 | 94.1 | 98.0 | 96.2 | 75.0 | 81.8 | 79.0 | 84.1 | 87.7 | 94.8 | 92.4 |
| **Child's sex** | | | | | | | | | | | | | | | | | | | | | | | | | | | | |
| Male | 88.9 | 89.4 | 91.5 | 94.7 | 96.2 | 97.8 | 97.0 | 71.4 | 76.2 | 74.2 | 84.3 | 89.6 | 94.0 | 92.0 | 72.4 | 67.1 | 73.6 | 86.2 | 90.6 | 94.3 | 92.9 | 75.7 | 78.8 | 76.1 | 82.0 | 84.9 | 90.8 | 87.6 |
| Female | 83.0 | 86.8 | 88.9 | 92.5 | 96.0 | 96.6 | 97.2 | 64.4 | 72.9 | 69.7 | 81.5 | 89.3 | 92.1 | 92.9 | 65.3 | 62.6 | 69.6 | 83.8 | 90.2 | 92.4 | 93.2 | 69.5 | 74.7 | 71.5 | 78.2 | 84.6 | 88.7 | 88.3 |
| **Current age of child (in years)** | | | | | | | | | | | | | | | | | | | | | | | | | | | | |
| 1 | 85.4 | 86.2 | 91.0 | 93.4 | 96.8 | 97.8 | 97.9 | 66.2 | 69.5 | 72.1 | 81.0 | 91.1 | 93.4 | 91.3 | 67.1 | 62.6 | 70.9 | 82.3 | 90.8 | 93.4 | 91.5 | 69.0 | 70.1 | 70.8 | 75.7 | 83.1 | 87.6 | 86.2 |
| 2 | 86.6 | 89.7 | 90.9 | 93.4 | 97.3 | 96.9 | 97.4 | 69.7 | 76.0 | 72.9 | 83.5 | 91.5 | 92.5 | 93.6 | 70.6 | 65.6 | 72.8 | 85.5 | 92.4 | 92.9 | 94.3 | 76.2 | 79.7 | 75.3 | 81.0 | 88.1 | 89.6 | 88.3 |
| 3 |  | 89.0 | 89.6 | 94.5 | 95.6 | 97.1 | 96.8 |  | 76.8 | 70.7 | 83.5 | 87.5 | 92.5 | 92.4 |  | 66.3 | 71.2 | 86.1 | 89.2 | 93.2 | 93.5 |  | 78.1 | 72.7 | 81.4 | 84.0 | 90.3 | 89.4 |
| 4 |  | 87.6 | 89.4 | 93.2 | 94.7 | 96.9 | 96.1 |  | 75.7 | 72.3 | 83.6 | 87.8 | 93.9 | 92.5 |  | 64.7 | 71.7 | 86.1 | 89.2 | 94.0 | 92.9 |  | 79.0 | 76.6 | 82.2 | 83.8 | 91.2 | 88.1 |
| **Size of child at birth** | | | | | | | | | | | | | | | | | | | | | | | | | | | | |
| Very large |  |  | 94.6 |  |  | 98.4 | 99.1 |  |  | 82.3 |  |  | 95.0 | 91.4 |  |  | 80.1 |  |  | 95.8 | 91.4 |  |  | 85.0 |  |  | 91.1 | 89.6 |
| Larger than average |  |  | 91.4 |  |  | 97.3 | 97.8 |  |  | 73.6 |  |  | 92.9 | 90.3 |  |  | 72.7 |  |  | 92.9 | 90.2 |  |  | 75.1 |  |  | 89.1 | 87.9 |
| Average |  |  | 89.9 |  |  | 97.4 | 97.7 |  |  | 72.4 |  |  | 93.4 | 93.6 |  |  | 72.1 |  |  | 93.8 | 93.6 |  |  | 74.2 |  |  | 90.2 | 87.7 |
| Smaller than average |  |  | 90.0 |  |  | 96.7 | 98.2 |  |  | 68.9 |  |  | 92.4 | 89.0 |  |  | 70.3 |  |  | 92.8 | 92.4 |  |  | 70.2 |  |  | 88.6 | 84.6 |
| Very small |  |  | 90.3 |  |  | 94.7 | 96.3 |  |  | 66.0 |  |  | 90.0 | 91.2 |  |  | 62.9 |  |  | 89.7 | 91.9 |  |  | 70.8 |  |  | 87.2 | 86.0 |
| **Birth order number** | | | | | | | | | | | | | | | | | | | | | | | | | | | | |
| 1 | 88.8 | 91.9 | 94.5 | 96.7 | 98.5 | 98.8 | 98.6 | 72.1 | 77.5 | 76.0 | 87.6 | 92.6 | 95.2 | 94.1 | 73.3 | 68.4 | 75.4 | 89.9 | 93.7 | 95.2 | 94.5 | 77.7 | 80.1 | 77.3 | 83.6 | 88.1 | 92.2 | 90.4 |
| 2 | 85.4 | 91.4 | 92.8 | 95.1 | 97.1 | 97.8 | 98.0 | 69.8 | 77.9 | 75.9 | 84.8 | 92.4 | 94.4 | 94.2 | 70.7 | 67.8 | 74.8 | 86.6 | 93.1 | 94.7 | 94.9 | 73.3 | 80.9 | 77.3 | 82.3 | 87.5 | 91.2 | 89.5 |
| 3 | 87.0 | 86.6 | 91.9 | 93.2 | 95.2 | 97.1 | 96.0 | 65.2 | 73.6 | 73.4 | 83.9 | 88.1 | 92.3 | 92.5 | 67.6 | 62.8 | 73.4 | 85.5 | 88.9 | 92.5 | 92.8 | 72.0 | 75.3 | 74.4 | 81.4 | 82.2 | 89.1 | 87.8 |
| 4 | 84.1 | 83.6 | 83.0 | 89.5 | 92.4 | 93.4 | 93.0 | 65.4 | 70.5 | 64.1 | 75.8 | 83.1 | 87.7 | 85.0 | 65.6 | 60.9 | 64.3 | 78.3 | 84.1 | 88.7 | 86.4 | 69.2 | 72.0 | 67.4 | 73.8 | 78.9 | 83.5 | 79.4 |
| **Skilled birth attendant** | | | | | | | | | | | | | | | | | | | | | | | | | | | | |
| No | 86.5 | 90.2 | 91.5 | 93.1 | 95.8 | 97.0 | 97.6 | 68.4 | 78.9 | 73.9 | 82.1 | 89.2 | 92.7 | 92.1 | 69.6 | 66.4 | 72.6 | 84.2 | 90.2 | 93.1 | 92.7 | 74.6 | 81.3 | 76.8 | 79.4 | 84.3 | 89.3 | 87.8 |
| Yes | 85.7 | 86.7 | 89.3 | 96.6 | 98.3 | 98.2 | 98.3 | 67.7 | 71.6 | 70.5 | 87.4 | 91.6 | 95.7 | 94.4 | 68.5 | 63.8 | 71.0 | 89.5 | 92.2 | 95.7 | 94.5 | 71.2 | 73.6 | 71.6 | 84.2 | 87.9 | 93.0 | 82.6 |
| **Having media exposure** | | | | | | | | | | | | | | | | | | | | | | | | | | | | |
| No | 83.9 | 86.1 | 88.5 | 92.5 | 95.0 | 96.0 | 96.3 | 64.6 | 71.6 | 68.2 | 80.1 | 87.0 | 91.1 | 90.4 | 65.8 | 62.9 | 68.6 | 82.3 | 88.1 | 91.4 | 91.2 | 68.8 | 74.2 | 69.6 | 76.9 | 82.1 | 87.5 | 85.1 |
| Yes | 91.8 | 92.4 | 93.5 | 95.3 | 98.0 | 99.0 | 98.3 | 77.3 | 80.9 | 79.0 | 87.3 | 93.8 | 96.2 | 95.4 | 77.6 | 69.0 | 77.3 | 89.1 | 94.4 | 96.6 | 95.7 | 83.2 | 82.3 | 81.8 | 85.0 | 89.3 | 93.5 | 92.0 |

**Supplementary Table 2:** Chi-square test results for measuring association among different characteristics and vaccination received by children aged 06-59 months, BDHS-2014

| **Characteristics** | **BCG*** | **DTP/ Pentavalent*** | **Polio*** | **Measles*** |
| --- | --- | --- | --- | --- |
| Place of residence | 0.025 | 0.075 | 0.036 | 0.013 |
| Region | <0.001 | <0.001 | <0.001 | <0.001 |
| Religion | 0.015 | 0.019 | 0.022 | <0.001 |
| Mother's age at 1st birth | 0.363 | 0.013 | 0.041 | <0.001 |
| Mother’s education | <0.001 | <0.001 | <0.001 | <0.001 |
| Father’s education | <0.001 | <0.001 | <0.001 | <0.001 |
| Mother’s working status | 0.203 | 0.593 | 0.676 | 0.024 |
| Father’s occupation | 0.006 | <0.001 | 0.001 | <0.001 |
| Wealth index | <0.001 | <0.001 | <0.001 | <0.001 |
| Delivery place | <0.001 | <0.001 | <0.001 | <0.001 |
| Child’s sex | 0.920 | 0.525 | 0.388 | 0.886 |
| Current age of child (in years) | 0.455 | 0.018 | 0.022 | 0.002 |
| Size of child at birth | 0.434 | 0.010 | 0.030 | 0.030 |
| Birth order number | <0.001 | <0.001 | <0.001 | <0.001 |
| Skilled birth attendant | 0.477 | 0.806 | 0.993 | 0.040 |
| Having media exposure | <0.001 | <0.001 | <0.001 | <0.001 |

***Only p-value is shown in the table**
